# Development and external validation of multiple machine learning-based models for breast cancer-specific survival prediction in postoperative patients with invasive breast cancer: a study based on the SEER database and an external cohort

**DOI:** 10.1186/s12885-026-16166-0

**Published:** 2026-05-30

**Authors:** ShengSheng Liu, JiaHui Liu, Dan Li, QinGuo Mo

**Affiliations:** 1 Guangxi Medical University Cancer Hospital, Nanning, Guangxi 530021 China; 2 The First People’s Hospital of Yulin, 495 JiaoYu Middle Road, Yuzhou District, Yulin, 537000 China; 3 Guangxi Medical University, Nanning, Guangxi 530021 China

**Keywords:** Invasive breast cancer (IBC), Breast cancer-specific survival (BCSS), The Surveillance, Epidemiology, and End Results program (SEER), Machine learning, Prognostic model

## Abstract

**Background:**

Patients with invasive breast cancer (IBC) make up most breast cancer cases and exhibit significant heterogeneity. Therefore, it is essential to construct an effective model to estimate long-term postoperative breast cancer-specific survival (BCSS).

**Methods:**

We used data from the Surveillance, Epidemiology, and End Results (SEER) database during 2010–2020, alongside recruiting an external cohort from Guangxi Medical University Cancer Hospital (GMUCH). We constructed four prediction models (Random Survival Forest [RSF], Survival Gradient Boosting Machine [Survival-GBM], Survival Extreme Gradient Boosting [Survival-XGBoost], and Least Absolute Shrinkage and Selection Operator-Cox proportional hazards model [LASSO-Cox]) to predict 3-, 5-, 7-, and 10-year BCSS in patients with IBC.

**Results:**

The RSF model developed in this study exhibited outstanding predictive performance, with a C-index of 0.824 (95% CI: 0.817–0.831) in the training set, 0.689 (95% CI: 0.670–0.707) in the internal validation set, and 0.716 (95% CI: 0.649–0.772) in the external validation set—outperforming its counterparts. Time-dependent Brier scores confirmed the model’s excellent calibration and high predictive accuracy. Decision curve analysis (DCA) further confirmed the model’s stable clinical utility, while Shapley Additive Explanations (SHAP) diagrams quantified the importance of prognostic features. Additionally, the RSF model demonstrated strong efficacy in stratifying patients into distinct BCSS risk subgroups.

**Conclusions:**

In conclusion, this study developed an optimal prognostic model for predicting the long-term BCSS of IBC patients, which provides critical support for risk stratification in the clinical management of IBC patients.

## Introduction

Over the past decades, breast cancer represents the world’s most prevalent malignant tumor and constitutes the second major reason for female cancer mortality [1]. Moreover, invasive breast cancer (IBC) displays prominent heterogeneity accompanied by divergent clinical prognoses, determined by tumor biology, treatment regimens, and health socioeconomic factors [2]. Thus precise, individualized predictions do not merely direct clinical treatment and follow-up arrangements but also ease the inherent prognostic uncertainty that burdens cancer patients in their post-diagnosis journey [3]. 

Traditional prognostic models offer clear interpretability and survival differences between subgroups, and therefore serve as the clinical “gold standard.” Nevertheless, although some optimized traditional models (e.g., extended TNM staging and nomograms) have incorporated interaction terms and multivariate inputs, most conventional models are based on linear assumptions and exhibit limited capacity in identifying complex nonlinear interactions among multiple clinical factors, high-dimensional data mining and precise individualized prognostic prediction [4, 5]. 

Machine learning demonstrates promising applicability across diverse clinical fields [6], including breast cancer prognosis prediction [7]. These models enable outcome prediction from clinical and tumoral covariates overlooked by classic models. [4]. A study performed by Weathers demonstrates that Random Survival Forests (RSF) exhibits superior performance to Cox proportional hazards model (CPH) and conditional inference forests (CIF) in the modeling of survival data [8]. In 2022, Huang et al. developed a model using least absolute shrinkage and selection operator (LASSO) regression to predict 3-year and 5-year survival outcomes in young breast cancer (YBC) patients [9]. Li et al. established an effective predictive model specifically for advanced breast cancer based on Extreme Gradient Boosting (XGBoost) [10]. As a popular ensemble learning algorithm，Gradient Boosting Machine (GBM) combines multiple decision trees via gradient descent boost model performance and exhibits exceptional efficacy in solving complex predictive tasks [11]. However, a recent systematic review of 922 models revealed that most clinical prediction models are hampered by insufficient reporting, methodological deficiencies, or elevated risk of bias, which indicates substantial room for further model development [12]. Additionally, most of these models exhibit suboptimal performance in external validation cohorts [13]. Another limitation of machine learning models is that they tend to generate “black-box” predictions [14, 15], and while Shapley Additive Explanations (SHAP) analysis can help demystify these predictions by quantifying feature importance and effect directionality, it cannot fully achieve complete mechanistic transparency.

We collected the variable features from the Surveillance, Epidemiology, and End Results (SEER) database and developed four models (Lasso-Cox, GBM, RSF, XGBoost) to predict the breast cancer-specific survival (BCSS) of IBC patients at 3, 5, 7, and 10 years. We externally validated the four models using an independent cohort from Guangxi Medical University Cancer Hospital (GMUCH).

## Materials and methods

### Data sources and study population

#### SEER database data

**Fig. 1:**
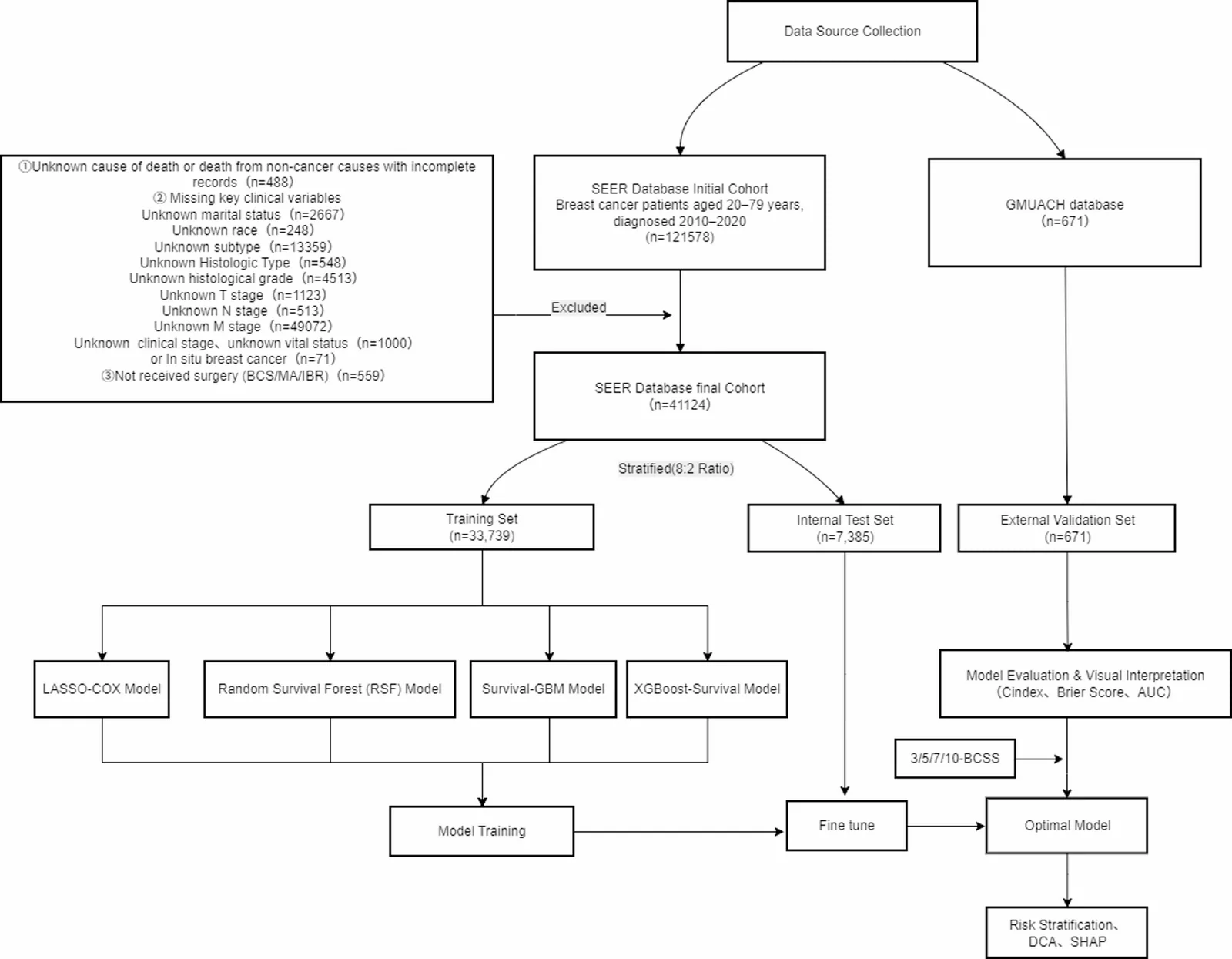
Flow chart of this study. Legend: SEER, Surveillance, Epidemiology, and End Results; GMUACH, Guangxi Medical University Affiliated Cancer Hospital; BCSS, breast cancer-specific survival; XGBoost, Extreme Gradient Boosting; AUC, area under the curve; DCA, decision curve analysis; SHAP, SHapley Additive exPlanations; BCS, breast-conserving surgery; MA, mastectomy; IBR, immediate breast reconstruction

SEER*Stat (version 8.4.2) was used to collect patient data from the SEER dataset. The open-access SEER database provided our original data, with records obtained from SEER 17 registries (2000–2020). Inclusion standards: ① Pathologically confirmed IBC diagnosed between 2010 and 2020. ② Aged 20–79 years. Exclusion criteria were: ① Undetermined/non-cancer death with incomplete records; ② Missing key variables such as marital status, race, molecular subtype, histological grade and type, TNM stage or breast cancer in situ; ③ Not received breast-conserving surgery (BCS), mastectomy (MA), or immediate breast reconstruction (IBR);. A total of 41,124 postoperative breast cancer patients were extracted from the SEER database, and stratified into a training set (*n* = 33,739) and an internal test set (*n* = 7,385) at an 8:2 ratio by outcome and clinical stage. Figure 1 illustrates the flowchart of this study.

#### External validation set data

A total of 671 IBC patients who received surgery at GMUCH (2010–2020) formed the external cohort. Although this cohort differed from the SEER database in geographic and demographic features, the two cohorts adopted the same inclusion and exclusion criteria. Case selection was confirmed by two senior oncologists through electronic medical record retrieval + manual review.

#### Ethical approval

Approved by the GMUCH Institutional Review Board (IRB, No. 2025041), this retrospective study waived informed consent due to anonymized datasets. Publicly available SEER data meets ethical requirements. All patients received standardized follow-up to determine survival status and cause of death, with the final assessment completed in December 2023. 

### Variables

Sixteen variables were enrolled in our study: marital situation, age, race, gender, AJCC stage, TNM stage, histologic grade, pathological classification, molecular subtype, percentage of metastatic lymph nodes, surgical type, chemotherapy, radiotherapy, and LODDS (log odds of metastatic), a continuous indicator reflecting individual lymph node metastatic burden calculated as the log ratio of positive lymph nodes to negative.

Cutoff thresholds of lymph node positive ratio and LODDS index were calculated via the X-tile package operated within R, a validated outcome-based algorithm for continuous variable dichotomization in survival analysis [16]. Although categorization of continuous variables may slightly reduce statistical efficiency, it greatly enhances clinical interpretability and applicability of the prognostic model, which is in accordance with the TRIPOD reporting guidelines [17]. 

### Model construction

#### LASSO-COX Model

**Fig. 2:**
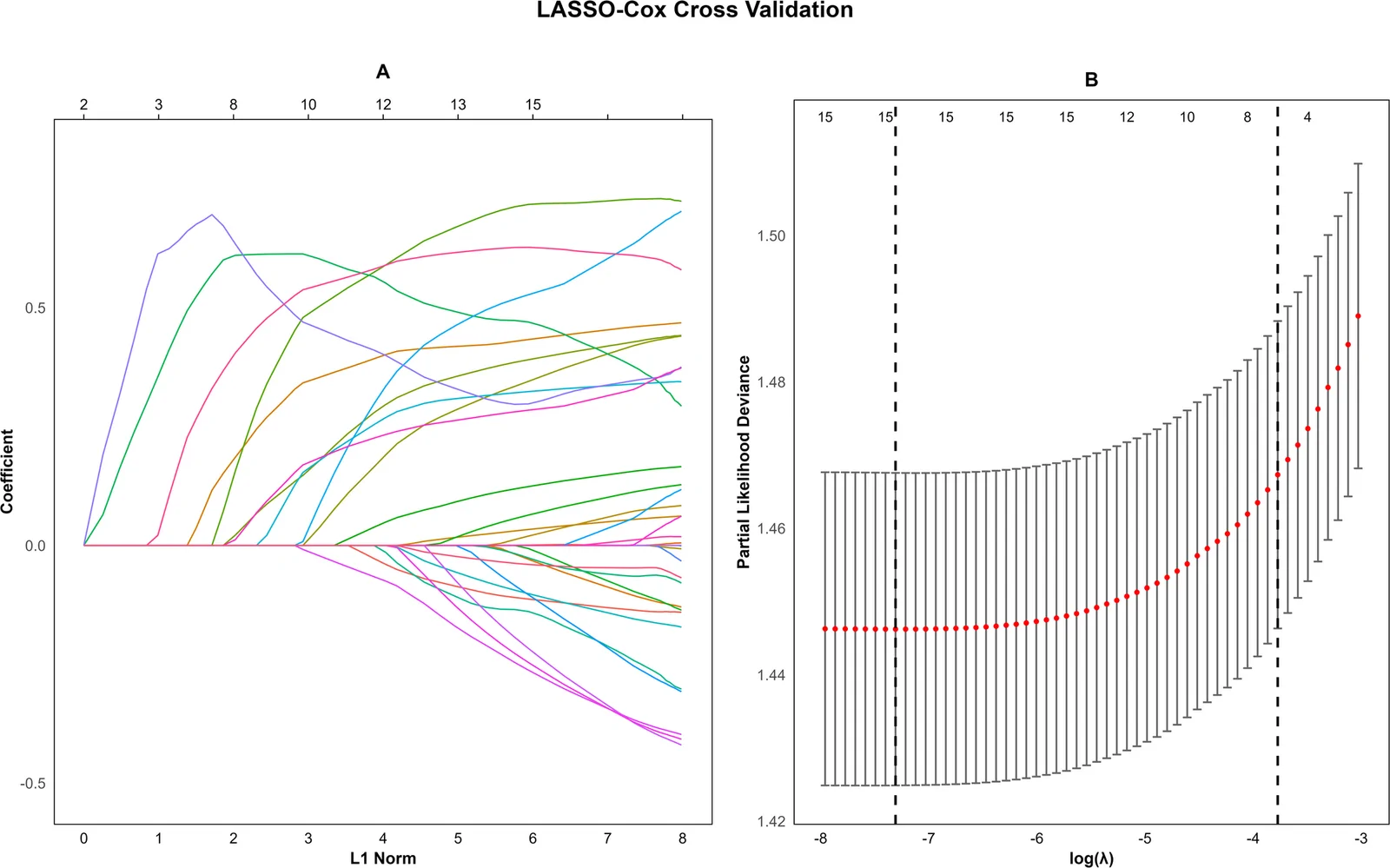
Cross-validation results of the LASSO-Cox model. Legend: (A) Coefficient trajectory of features with increasing L1 norm (different colors = distinct features); (B) Partial likelihood deviance across log(λ) values (red dots = optimal λ; dashed lines = λ at minimum deviance and 1-standard error rule)

LASSO regression (alpha = 1) was used for feature selection in the training set under the Cox proportional hazards framework (family = “cox”) via 10-fold cross-validation (nfolds = 10). The regularization parameter λ was optimized over a range of values (10⁻⁵ to 10²), and λ.min (minimum cross-validation error) was selected for feature screening to achieve optimal predictive performance (Figure 2). Of note, λ.min corresponds to a relatively complex model with higher potential overfitting risk, and λ.1se is the standard conservative criterion for anti-overfitting in LASSO regression. Filtered predictors were fitted into Cox regression for proportional hazard evaluation, and Schoenfeld residual inspection was performed to validate the proportional hazards premise.

#### Random survival forest (RSF) model

The RSF model was built with postoperative BCSS time/cancer-specific death as outcomes and 16 features as predictors.Prior to model construction, patients with missing key clinical variables (marital status, race, molecular subtype, histological grade and type, TNM stage, etc.) were strictly excluded according to the predefined exclusion criteria.Hyperparameters were optimized via 10-fold cross-validation over ranges (ntree: 100–1000, nodesize: 1–20), with final values: ntree = 500, nodesize = 10;for residual missing values in predictive covariates among the included patients, missing values were imputed using na.action = “na.impute” (median and mode imputation for continuous and categorical variables, respectively), and seed = 123 ensured reproducibility.

#### Survival-GBM model

A gradient boosting machine (GBM) model was constructed using the Cox partial likelihood function as the objective function. Hyperparameters were optimized via 10-fold cross-validation (learning rate: 0.01–0.1, tree depth: 3–10, iterations: 100–1000, subsample ratio: 0.7–1.0). The final optimal hyperparameters were determined as follows: learning rate = 0.01, tree depth = 3, iterations = 1000 (optimal trees via cross-validation), subsample ratio = 1.0. Of note, a subsample ratio of 1.0 indicates that stochastic subsampling regularization was not adopted during tree construction. Although subsampling acts as a key anti-overfitting mechanism for GBM, cross-validation results demonstrated the optimal predictive performance at this upper boundary value.

#### XGBoost-survival model

The XGBoost survival model (objective: “survival: cox”, eval_metric: “cox-nloglik”) was built with hyperparameters optimized over ranges (eta: 0.01–0.08, max_depth: 3–8, subsample/colsample_bytree: 0.6–0.9); final values were eta = 0.01, max_depth = 3, subsample/colsample_bytree = 0.8 (seed = 123). Trained for 1000 maximum iterations, early stopping (50 rounds) selected the best iteration number (best_iteration) to mitigate overfitting.

### Model evaluation indicators

Discrimination: C-index (95% confidence interval [CI]) and time-dependent ROC curve/AUC value (3-, 5-, 7-, and 10-year) were used. A C-index ≥ 0.70 in the external validation set was considered to indicate good discriminative and generalization ability, according to the classic grading criteria for prognostic model performance [18]. 

Calibration: Brier score (3-, 5-, 7-, and 10-year) and calibration curves were used for calibration assessment. Time-dependent Brier score was calculated with the R package pec [19], with inverse probability censoring weighting (IPCW) adjustment for censored data. No scaling, normalization or outcome transformation was performed during calculation. A Brier score < 0.25 indicates predictive performance superior to the uninformative null model. Of note, this threshold represents a basic reference standard rather than good calibration performance. A difference ≤ 0.05 between the external validation set and training set indicated stable calibration.

Clinical utility: Decision Curve Analysis (DCA) was used to calculate clinical net benefits under different threshold probabilities, focusing on the 10%-30% interval related to clinical practice.

Feature importance: Regression coefficients (LASSO-COX), node impurity reduction/feature gain (machine learning models), and SHAP analysis (to visualize feature contribution and linear/non-linear associations) were used to assess the importance of prognostic features.

Feature-specific predictive effects were further quantified by mean absolute SHAP values (MASV) [20, 21], with explicit interpretation of effect directionality and clinical significance for both conventional staging factors and non-obvious prognostic predictors.

Risk stratification: The risk threshold was determined using the X-tile function in R software (fixed across all datasets). Patients were stratified into high-risk and low-risk groups based on this threshold, and Kaplan-Meier (KM) curves combined with log-rank tests were used to compare differences in BCSS between the two groups.

### Statistical analysis software

All analyses (data cleaning, descriptive statistics) were performed using R software (version 4.5.2). Continuous variables were converted to categorical variables via optimal cut-offs identified by the R X-tile function. Models were built with R packages (rms, glmnet, random Forest SRC, gbm, xgboost, pec) and visualized with ggplot2, survminer, DCAplots, SHAP-related packages. Original continuous variables were compared via Wilcoxon rank-sum test, and categorical variables (including converted ones) via χ²/Fisher’s exact test. Model performance was compared using Bootstrap (1000 repetitions), with *P* < 0.05 (two-sided) as statistically significant.

## Results

### Baseline characteristics of patients

A total of 41,795 patients were recruited into this study. Table 1 baseline characteristics of enrolled patients across three datasets.

**Table 1:** Distribution of baseline characteristics

Baseline Characteristics	Training Set (*n* = 33739)	Test Set (*n* = 7385)	Validation Set (*n* = 671)	*P*-value
Age group, n (%)				< 0.001
20–39 years (Group 1)	852 (2.5)	185 (2.5)	123 (18.3)	
40–59 years (Group 2)	11,524 (34.2)	2674 (36.2)	460 (68.6)	
60–79 years (Group 3)	21,363 (63.3)	4526 (61.3)	88 (13.1)	
Marital Status, n (%)				< 0.001
Married (including common law)	19,840 (58.8)	4274 (57.9)	652 (97.2)	
Separated/Divorced/Widowed	8817 (26.1)	1859 (25.2)	2 (0.3)	
Single (never married)/Unmarried/Domestic Partner	5082 (15.1)	1252 (17.0)	17 (2.5)	
Sex, n (%)				0.064
Female	33,406 (99.0)	7305 (98.9)	670 (99.9)	
Male	333 (1.0)	80 (1.1)	1 (0.1)	
Race, n (%)				< 0.001
American Indian/Alaska Native	198 (0.6)	39 (0.5)	0 (0.0)	
Asian	2697 (8.0)	654 (8.9)	671 (100.0)	
Black	3194 (9.5)	811 (11.0)	0 (0.0)	
White	27,650 (82.0)	5881 (79.6)	0 (0.0)	
Histologic Type, n (%)				< 0.001
Infiltrating Ductal Carcinoma	24,932 (73.9)	5309 (71.9)	490 (73.0)	
Infiltrating Lobular Carcinoma	5098 (15.1)	1188 (16.1)	130 (19.4)	
Invasive Breast Carcinoma of Special Type	3709 (11.0)	888 (12.0)	51 (7.6)	
Grade, n (%)				< 0.001
Grade I	9118 (27.0)	1850 (25.1)	58 (8.6)	
Grade II	15,649 (46.4)	3411 (46.2)	453 (67.5)	
Grade III	8972 (26.6)	2124 (28.8)	160 (23.8)	
Molecular Subtype, n (%)				< 0.001
HR-/HER2-	3499 (10.4)	805 (10.9)	72 (10.7)	
HR-/HER2+	1051 (3.1)	228 (3.1)	107 (15.9)	
HR+/HER2-	26,400 (78.2)	5640 (76.4)	354 (52.8)	
HR+/HER2+	2789 (8.3)	712 (9.6)	138 (20.6)	
T Stage, n (%)				< 0.001
T0-T1	22,793 (67.6)	4613 (62.5)	145 (21.6)	
T2	8957 (26.5)	2242 (30.4)	391 (58.3)	
T3	1494 (4.4)	395 (5.3)	71 (10.6)	
T4	495 (1.5)	135 (1.8)	64 (9.5)	
N Stage, n (%)				< 0.001
N0	24,570 (72.8)	5160 (69.9)	230 (34.3)	
N1	6756 (20.0)	1624 (22.0)	187 (27.9)	
N2	1534 (4.5)	389 (5.3)	152 (22.7)	
N3	879 (2.6)	212 (2.9)	102 (15.2)	
M Stage, n (%)				< 0.001
M0	33,530 (99.4)	7331 (99.3)	640 (95.4)	
M1	209 (0.6)	54 (0.7)	31 (4.6)	
Clinical Stage, n (%)				< 0.001
Stage I	18,993 (56.3)	3757 (50.9)	51 (7.6)	
Stage II	13,315 (39.5)	3269 (44.3)	344 (51.3)	
Stage III	1222 (3.6)	305 (4.1)	245 (36.5)	
Stage IV	209 (0.6)	54 (0.7)	31 (4.6)	
Node Rate, n (%)				< 0.001
>0.03	8912 (26.4)	2152 (29.1)	441 (65.7)	
0	24,778 (73.4)	5219 (70.7)	230 (34.3)	
0-0.03	49 (0.1)	14 (0.2)	0 (0.0)	
LODDS Group, n (%)				< 0.001
(-0.88, 1.24]	4161 (12.3)	934 (12.6)	158 (23.5)	
<=-0.88	28,745 (85.2)	6234 (84.4)	457 (68.1)	
>1.24	833 (2.5)	217 (2.9)	56 (8.3)	
Surgery, n (%)				< 0.001
BCS (Breast-Conserving Surgery)	17,326 (51.4)	3370 (45.6)	114 (17.0)	
IBR (Immediate Breast Reconstruction)	5423 (16.1)	1350 (18.3)	78 (11.6)	
MA (Mastectomy)	10,990 (32.6)	2665 (36.1)	479 (71.4)	
Chemotherapy, n (%)				< 0.001
No/Unknown	20,832 (61.7)	4182 (56.6)	25 (3.7)	
Yes	12,907 (38.3)	3203 (43.4)	646 (96.3)	
Radiation, n (%)				< 0.001
No/Unknown	9357 (27.7)	2249 (30.5)	297 (44.3)	
Refused	673 (2.0)	166 (2.2)	35 (5.2)	
Yes	23,709 (70.3)	4970 (67.3)	339 (50.5)	

1. Categorical variables were compared using the chi-square test. P<0.05 was considered statistically significant. 2. Indentation indicates subcategories of the main baseline characteristics for clear readability. 3. The table follows the reporting standards for machine learning dataset partitioning in academic papers. 4. Total sample size: 41795 (Training Set + Test Set + Validation Set). 5. LODDS: Log Odds of Positive Lymph Nodes (positive lymph node log odds)

There were significant differences in demographic features (age, marital status, race), pathological indicators (histologic type and grade, molecular subtype), stage parameters (TNM stage, clinical stage), lymph node-related indices (Node Rate, LODDS), and treatment modalities (surgery type, chemotherapy, radiation) between the GMUCH cohort and the SEER cohort (all *P* < 0.001).Relative to the SEER cohort, the GMUCH cohort was dominated by young and middle-aged married Asian patients, whereas the SEER cohort mainly consisted of elderly patients with diverse marital statuses and a predominantly White racial composition. In terms of pathological features, the GMUCH cohort had a higher proportion of infiltrating lobular carcinoma, Grade II tumors, HR-/HER2 + and HR+/HER2 + subtypes, and a lower proportion of Grade I tumors and HR+/HER2- subtype. For stage and lymph node indicators, the GMUACH cohort was characterized by more advanced T/N/M stages, fewer early clinical stages, more lymph node-positive cases, and higher lymph node-related risk levels. Regarding treatment, the GMUACH cohort was dominated by mastectomy with a lower proportion of breast-conserving surgery, and a higher chemotherapy rate but a lower radiation rate (with more cases of unknown/no radiation) relative to the SEER cohort. Nevertheless, the two cohorts did not differ significantly in gender composition (*P* = 0.064), with the vast majority of patients being female. The significant racial difference (the external validation set was entirely Asian) is an inherent regional attribute.

### Comparison of model performance (Discrimination)

**Fig. 3:**
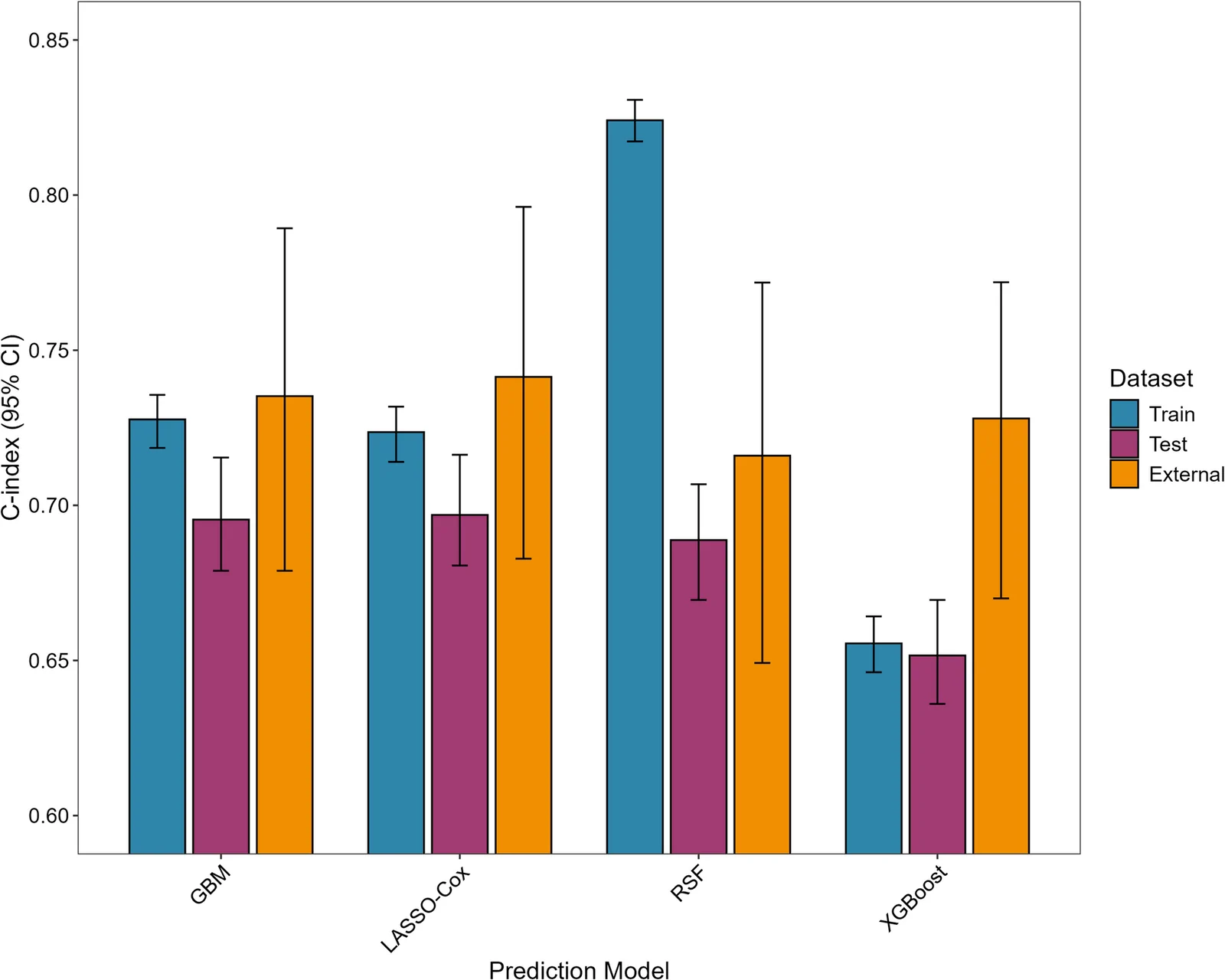
Comparison of C-index Values (with 95% CI) Among Four Survival Prediction Models in Training, Internal Test, and External Validation Datasets Legend: Blue bars = Training dataset; Purple bars = Internal test dataset; Orange bars = External validation dataset; Dashed red line = Reference threshold (C-index=0.5, random prediction). Error bars = 95% CI of C-index values.

Four models were assessed for discrimination in internal and external validation sets (Table 2 and Fig. 3). RSF showed favorable generalizability despite not being top in external C-index; LASSO-Cox had the highest C-index, and XGBoost performed poorly.

**Table 2:** Comparison of C-index and 95% Confidence Interval of Each Model on Different Datasets

Model	C-Index (Train, 95%CI)	C-Index (Test, 95%CI)	C-Index (External, 95%CI)
LASSO-Cox	0.724 (0.714–0.732)	0.697 (0.681–0.716)	0.741 (0.683–0.796)
RSF	0.824 (0.817–0.831)	0.689 (0.670–0.707)	0.716 (0.649–0.772)
GBM	0.728 (0.719–0.736)	0.695 (0.679–0.715)	0.735 (0.679–0.789)
XGBoost	0.656 (0.646–0.664)	0.652 (0.636–0.670)	0.728 (0.670–0.772)

The C-index is used to evaluate the discriminative ability of survival prediction models, with a value range of 0.5-1.0. A value closer to 1 indicates stronger discriminative ability, while a value of 0.5 indicates no discriminative ability. Each cell in the table is formatted as “C-index (95% confidence interval)”, where the 95% confidence interval (95%CI) reflects the statistical stability of the C-index, and a narrower interval indicates more reliable results. The datasets include the training set (Train), test set (Test), and external validation set (External)

**Fig. 4:**
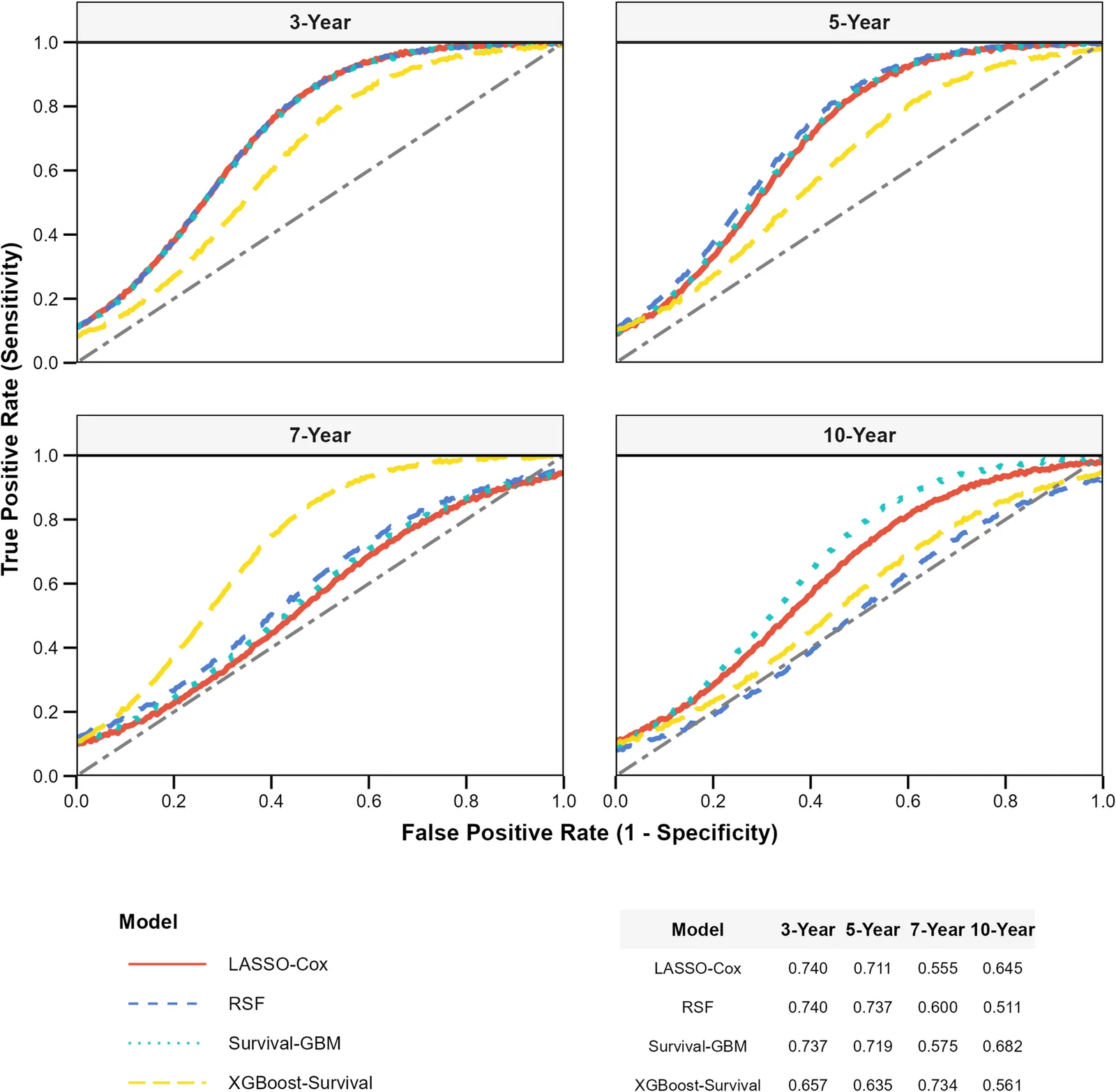
Time-dependent ROC curves of four models at 3-, 5-, 7-, and 10-year follow-up. Legend: Solid red line = LASSO-Cox; Dashed gray line = RSF; Dotted blue line = Survival-GBM; Dashed yellow line = XGBoost-Survival; Table inset = AUC values of each model at different follow-up years

Time-dependent AUC (Table 3 and Fig. 4) varied with time and dataset. RSF excelled in short-to-medium term; XGBoost and GBM had long-term advantages. All models had comparable AUC internally.

**Table 3:** Comparison of model performance (AUC Values)

Model	3-year AUC	5-year AUC	7-year AUC	10-year AUC
LASSO-Cox	0.740	0.711	0.555	0.646
RSF	0.740	0.737	0.600	0.511
GBM	0.737	0.719	0.575	0.682
XGBoost	0.657	0.635	0.734	0.561

AUC = Area under the time-dependent receiver operating characteristic curve, which reflects the discrimination ability of the model to distinguish between cancer-specific death and survival outcomes in postoperative breast cancer patients. A higher AUC value indicates better discrimination performance of the model

### Comparison of model performance (Calibration)

Calibration was assessed by Brier scores (Table 4; lower = better). RSF had optimal and stable calibration across all time points and validation sets, outperforming others. GBM was comparable to RSF externally; LASSO-Cox and XGBoost had higher scores without significant long-term deterioration.

**Table 4:** Comparison of model performance (Brier Score)

Model	3-year Brier score	5-year Brier score	7-year Brier score	10-year Brier score
LASSO-Cox	0.195	0.208	0.219	0.228
RSF	0.024	0.043	0.060	0.083
Survival-GBM	0.031	0.059	0.084	0.123
XGBoost-Survival	0.202	0.215	0.223	0.235

Brier score ranges from 0 to 1, with a lower value indicating better calibration of the model for postoperative breast cancer patients’ CSS prediction

The RSF model maintained stable calibration performance across both internal and external validation sets. The GBM model showed comparable calibration to the RSF model in the external validation set, while the LASSO-Cox and XGBoost models had relatively higher Brier scores without significant deterioration in long-term calibration.

In conclusion, through a systematic multi-dimensional evaluation framework encompassing discrimination, calibration, and clinical utility, we substantively demonstrate that the RSF model outperforms alternatives by achieving balanced short-to-medium term discriminative ability and robust calibration stability across internal and external validations. In contrast, other models exhibit inherent limitations, such as suboptimal overall discriminative performance (e.g., XGBoost) or inferior calibration stability, thereby justifying the selection of RSF as the optimal model for prognostic predictions.

### Feature importance analysis

Feature importance was evaluated using the Random Survival Forest (RSF) model, and SHAP was applied to clarify the prognostic mechanism and demystify the machine learning black box. To rigorously quantify the global predictive effect magnitude of each feature, we calculated mean absolute SHAP values (MASV), the standard quantitative metric for SHAP effect assessment [20, 21]. 

**Fig. 5:**
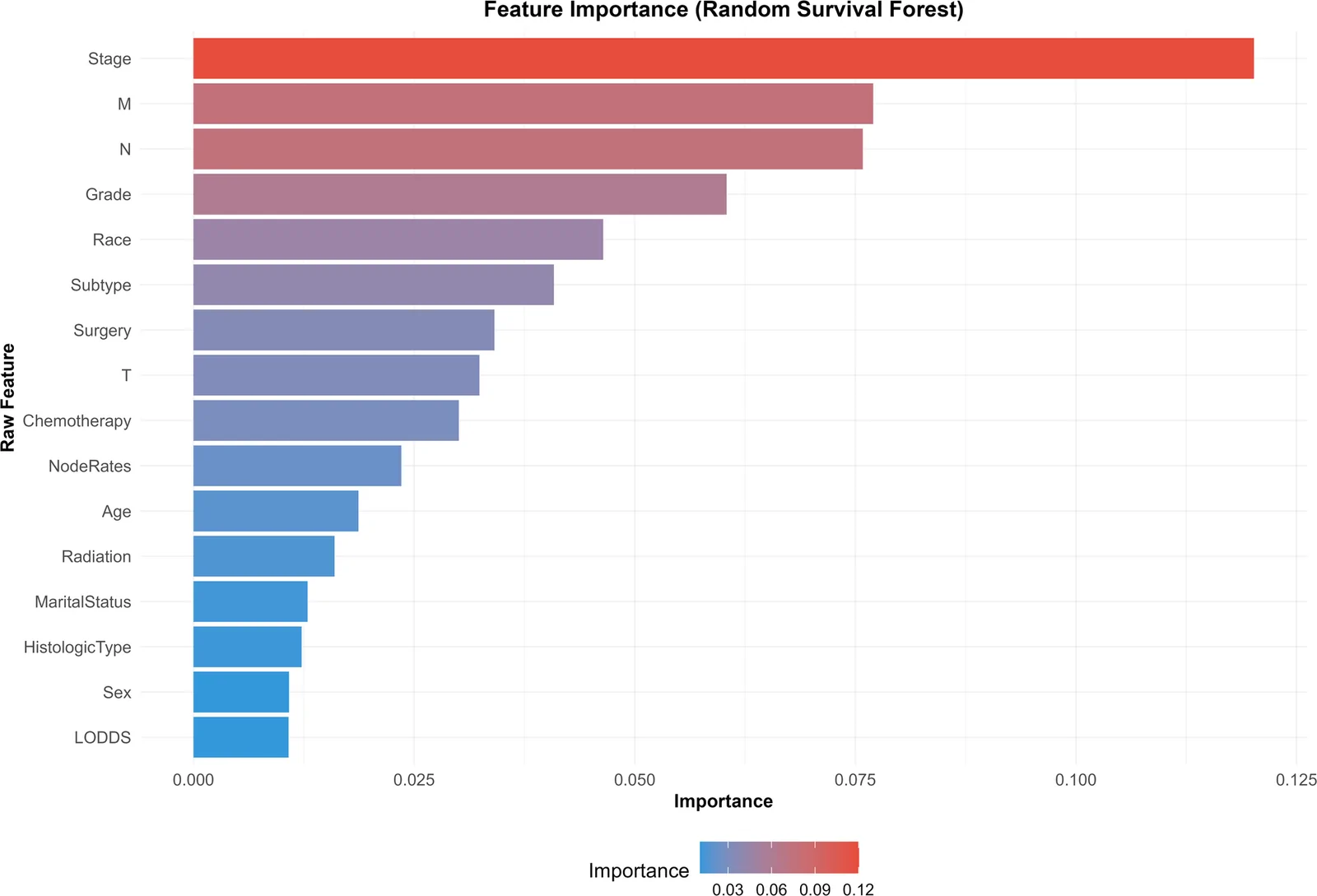
Feature importance ranking of the Random Survival Forest model. Legend: Bar length represents the importance score of each feature; Color gradient (from blue to red) corresponds to importance scores (1000 to 3000)

As shown in Fig. 5, the importance of all variables was quantitatively ranked by MASV: Stage presented the highest importance (MASV = 0.118), followed by M stage (MASV = 0.078), N stage (MASV = 0.078), and histological Grade (MASV = 0.062). In contrast, features including LODDS exhibited relatively low importance (MASV = 0.008).

According to the SHAP summary plot interpretation, advanced overall Stage, higher N stage, higher histological Grade and M stage correspond to SHAP values greater than 0.05, indicating that these factors significantly increase the risk of cancer-specific mortality. Beyond these well-established staging factors, we further analyzed non-obvious prognostic features including Race, molecular Subtype, and Surgery status. These variables showed heterogeneous SHAP effect distributions across patients, with both positive and negative contributions to cancer-specific mortality risk, reflecting their subgroup-specific prognostic value in breast cancer survival stratification.

### Risk stratification results

Patient risk stratification is critical for guiding clinical management. Patient risk scores were calculated with the RSF model, and the threshold was then used to separate patients into two risk cohorts.

**Fig. 6:**
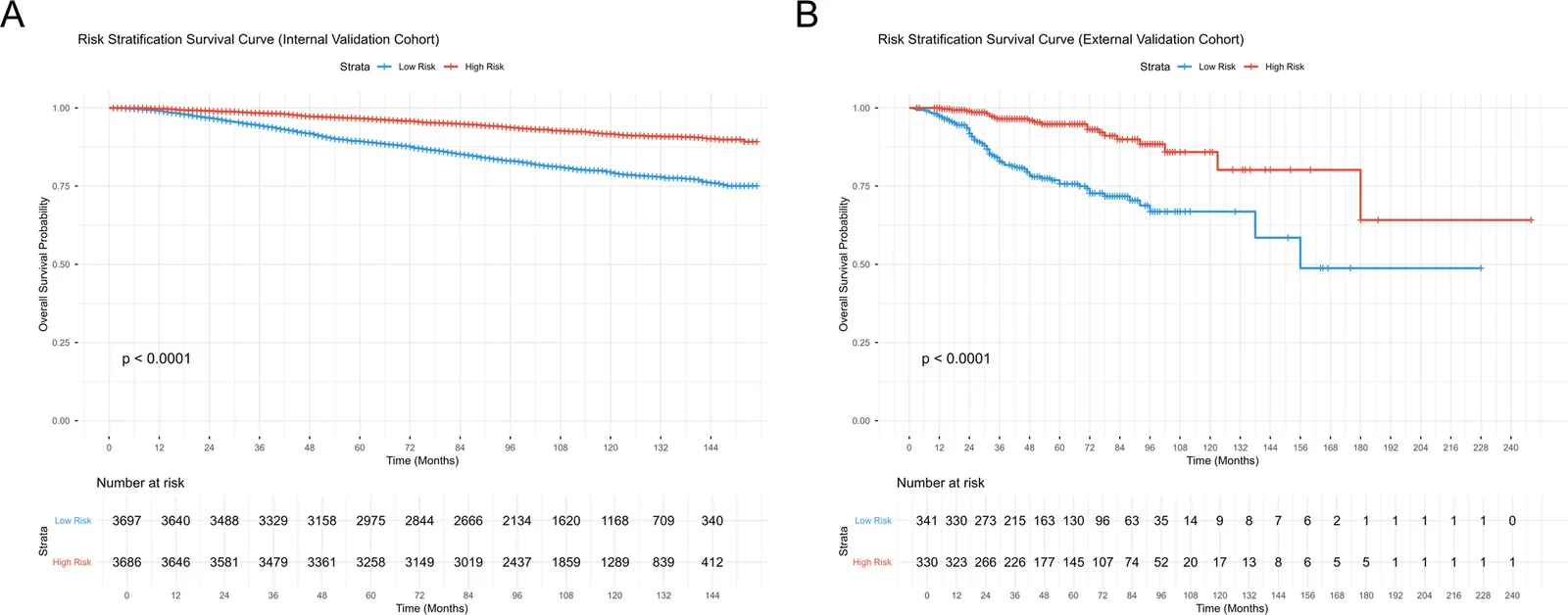
Risk stratification survival curve in validation cohorts. Legend: Blue = Low-risk group; Red = High-risk group; Note: (A) External validation cohort; (B) Internal validation cohort. Table below shows at-risk patient counts per group at different follow-up time points. log-rank test, p < 0.0001

Kaplan-Meier analysis and log-rank tests (Fig. 6A and B) showed significant differences between the two cohorts (log-rank *P* < 0.001).

## Discussion

### Summary of key research findings

Invasive breast cancer (IBC) accounts for the vast majority of breast cancer cases; however, IBC patients generally have poorer prognosis than those with ductal carcinoma in situ (DCIS) [22]. As a heterogeneous disease with variable outcomes, IBC requires precise, individualized predictions to support stratified clinical follow-up, prognostic counseling, and identification of high-risk candidates for clinical trials [23]. 

We constructed and evaluated four machine learning models (Lasso-Cox, GBM, RSF, XGBoost) for BCSS prediction in IBC patients. Consistent with our empirical results rather than general theoretical assumptions, the RSF model showed superior overall performance, highest discrimination in the training cohort, stable external discrimination and optimal calibration across distinct cohorts. Notably, the RSF model maintained stable performance across heterogeneous cohorts with distinct geographic and demographic backgrounds, thereby confirming its excellent generalization ability. Our specific SHAP quantitative results further revealed that race contributed minimal predictive weight to the RSF model, which minimally impacts its predictive performance and explains the model’s stability despite baseline discrepancies.

**Fig. 7:**
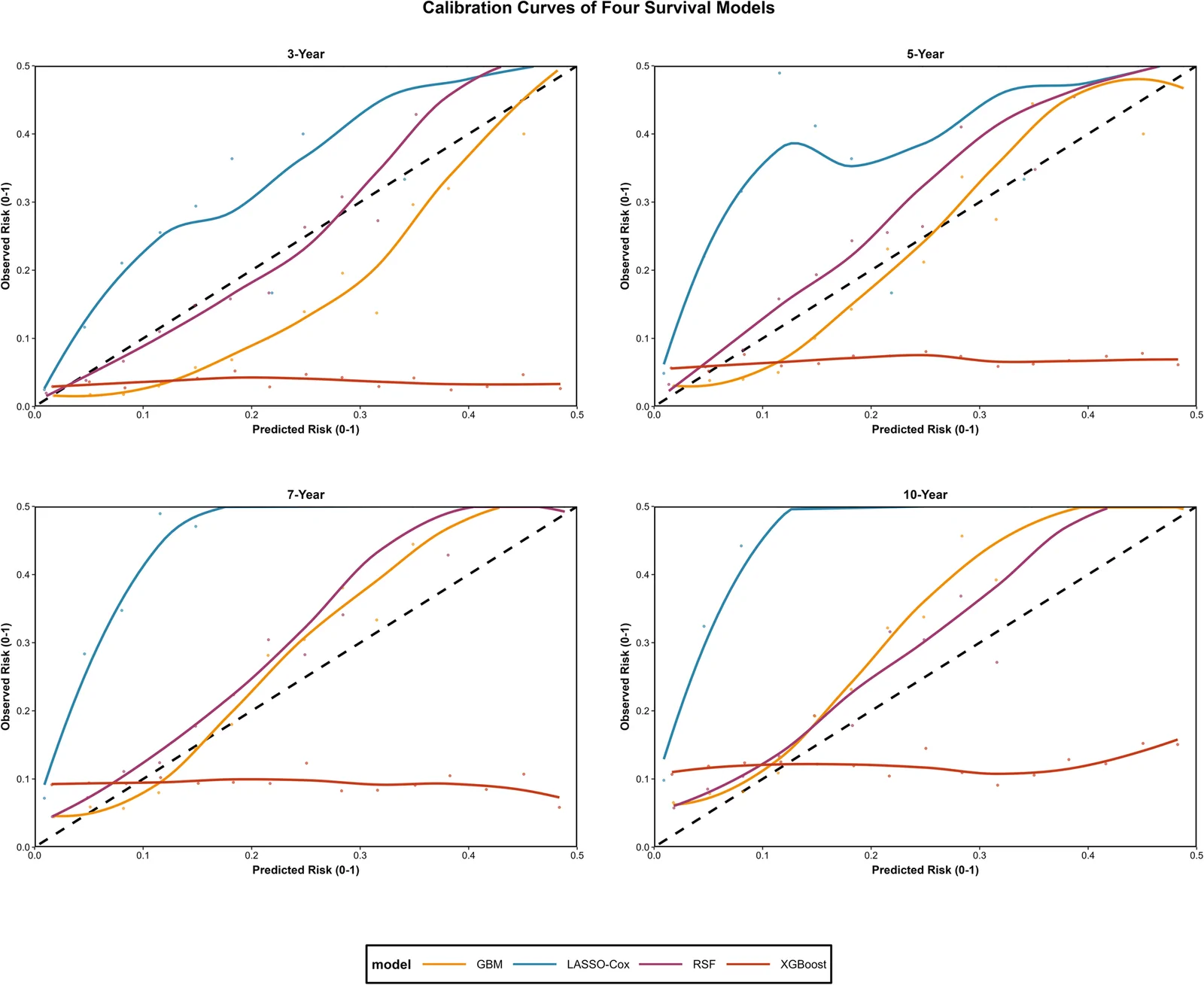
Calibration curves of four survival models for predicting 3-, 5-, 7-, and 10-year BCSS in postoperative invasive breast cancer patients. Legend: LASSO-Cox (blue); RSF (purple); GBM (orange); XGBoost (red); dashed black line = ideal calibration (perfect agreement between predicted and observed risks). Notes: Calibration curves assess the agreement between predicted risk (x-axis) and observed risk (y-axis) of breast cancer-specific survival (BCSS) for four survival models at 3-, 5-, 7-, and 10-year follow-up. A curve closer to the dashed line indicates better calibration performance

As visualized in the calibration curves (Fig. 7), which plot predicted versus observed BCSS risk at 3-, 5-, 7-, and 10-year follow-up, the RSF model maintained curves closest to the ideal diagonal reference line across all time horizons, indicating favourable concordance between predicted risks and observed rates. Similarly, the LASSO-Cox model showed good calibration overall, with only minor deviations at low predicted probabilities in the 5-year plot. In contrast, the GBM model exhibited time-dependent calibration: while its curves converged closely to the ideal line at 7- and 10-year follow-up, mild S-shaped deviations were observed at 3- and 5-year time points, reflecting a tendency to underestimate risk at low probabilities and overestimate at higher probabilities. Most notably, the XGBoost model demonstrated severe, persistent miscalibration across all time points, with its curve remaining far beneath the ideal line, suggesting systematic underestimation of BCSS risk.

Furthermore, this study also found that IBC patients in the cohort from GMUCH cohort were younger overall, with later AJCC clinical stages, greater numbers of axillary nodal metastases and more conservative surgical choices than SEER cohort counterparts. Notably, the distribution of molecular subtypes and pathological classifications in this cohort differed significantly from those in the SEER cohort. This aligns with prior investigations that compared the clinicopathological features and treatment modalities of breast cancer patients between China and West [24, 25]. 

The risk stratification results precisely distinguished high-risk and low-risk subgroups and showed stable performance in the external validation cohort. High-risk patients may potentially benefit from intensified adjuvant therapy (e.g., dose-dense chemotherapy, endocrine therapy, radiotherapy, targeted therapy). In contrast, low-risk patients may avoid unnecessary chemotherapy and receive extended follow-up intervals. These risk-stratified personalized strategies are exploratory model-based suggestions requiring prospective clinical validation, which may help reduce treatment-related toxicities and medical costs.

Our direct head-to-head comparison results verified the practical superiority of RSF over LASSO-Cox, GBM and XGBoost in our specific BCSS prediction scenario. RSF showed unique advantages in capturing non-linear prognostic interactions for heterogeneous breast cancer survival data.

**Fig. 8:**
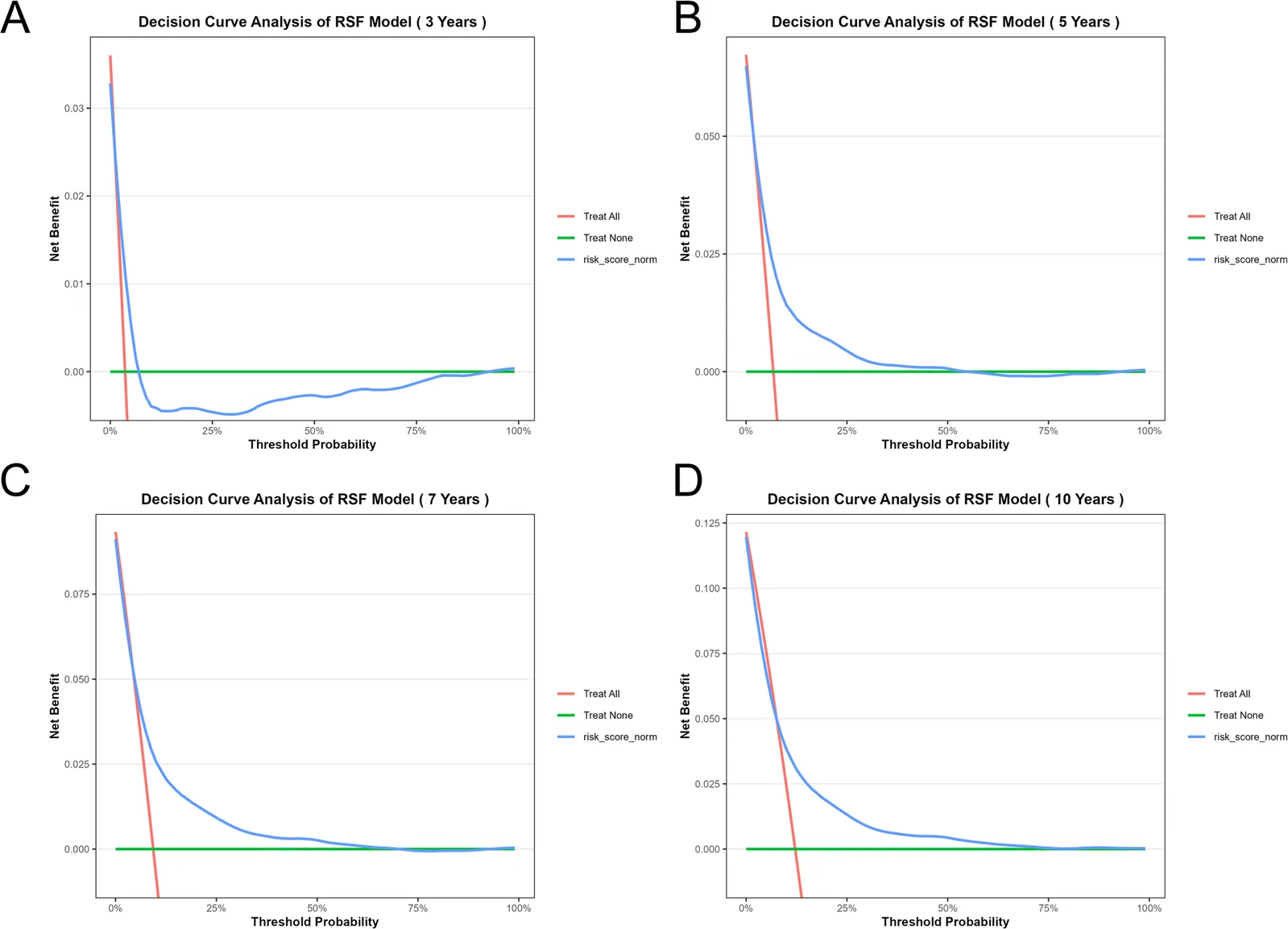
Decision Curve Analysis of the RSF Model for Predicting 3-, 5-, 7-, and 10-Year BCSS in Postoperative Invasive Breast Cancer (IBC) Patients. Legend: Red (Treat All) = Net benefit of treating all; Green (Treat None) = Net benefit of treating none; Blue (risk_score_norm) = Net benefit of RSF-guided decisions; Note: Decision curve analysis of the RSF model for 3-, 5-, 7-, and 10-year BCSS in postoperative IBC patients (**A**–**D**: 3-, 5-, 7-, 10-year BCSS, respectively). X-axis (Threshold Probability): Critical clinical decision threshold (acceptable event risk for clinicians/patients); Y-axis (Net Benefit): Clinical net benefit of model-guided decisions (higher = stronger utility)

Given that clinical validity outweighs statistical validity [26], DCA was adopted to assess clinical utility. Consistent with our observed data characteristics, the RSF model achieved favorable net benefit for 5-,7-,10-year BCSS prediction (Fig. 8). However, 3-year net benefit was limited by the extremely low 3-year BCSS event rate, which reduced outcome variability and restricted short-term model performance.

Although neural network models exhibit impressive performance, their inherent black-box nature remains a major barrier to clinical translation [27]. Our RSF model achieves a favorable balance between predictive performance and interpretability in this clinical setting. In addition, feature importance was quantitatively evaluated by mean absolute SHAP values (MASV). AJCC stage remained the dominant prognostic factor, followed by M stage, N stage, histological grade, race and molecular subtype. These quantitative findings provide deeper prognostic insights beyond qualitative consistency with prior studies.

### Comparison with existing studies

Although there exist models for breast cancer (BC) mortality (generally all-cause mortality), they are not suitable for all patients and their statistical methodologies are not the most powerful; even the only model identified as low-risk for bias suffers from insufficient sample size and unnecessary dichotomization of predictive variables [28]. In contrast, this study enrolled IBC patients aged 20–80 from the SEER database, which provides abundant samples to ensure population generalization and adequate model learning.

Consistent with previous RSF applications in inflammatory breast cancer [29], our RSF model showed excellent short-term (3/5-year) AUC performance; however, we specifically observed notable long-term (7–10-year) AUC deterioration in our cohort, which was likely attributed to declining event numbers and amplified unmeasured confounding during extended follow-up, a unique finding of our long-term follow-up data.

Despite prior studies reporting LODDS as an independent predictive indicator for invasive micropapillary carcinoma [30], it showed minimal importance in our models. The low feature importance of LODDS in this study (samples with < 10 dissected lymph nodes excluded) may be due to three factors: IBC population heterogeneity, strong collinearity with N stage, and the feature importance logic of ensemble models (RSF, XGBoost, GBM).

Tools like PREDICT Breast [31] and the Nottingham Prognostic Index (NPI) [32] are widely used to predict prognosis and guide postoperative treatment decisions in early-stage BC patients. However, external validation has shown that PREDICT and the NPI exhibit suboptimal performance in elderly women and certain high-risk subgroups [13, 33]. This highlights the need for stable model performance evaluation. Thus, we performed external validation to confirm the model’s generalizability. Furthermore, some previous studies included risk factors that require invasive detection (e.g., breast biopsy and genetic testing), which limits their widespread clinical application [34], the RSF model constructed in this study is developed based on routine clinical features, featuring lower cost and higher accessibility.

Notably, the low 3-year BCSS event rate limited short-term net benefit of the RSF model, and its 10-year discriminative performance declined markedly with limited long-term predictive reliability. Beyond conventional discriminative evaluation, multi-timepoint calibration curves (Fig. 8) provided intuitive visual evidence for the probabilistic performance of each model. Even with acceptable discriminative ability, GBM presented mild short-term miscalibration, whereas XGBoost displayed sustained and severe miscalibration throughout all follow-up periods, greatly weakening its clinical application value regardless of its statistical performance in discrimination. LODDS showed minimal prognostic importance, likely due to case exclusion and collinearity with N stage.

### Advantages of the study

This study focuses on postoperative invasive breast cancer (IBC) patients and BCSS prediction with robust population specificity, directly addressing the clinical need for personalized treatment in this cohort. We carried out a systematic comparison of four models, including the traditional regularized LASSO-Cox model and three advanced machine learning models (RSF, Survival-GBM, XGBoost-Survival), to ensure the objective identification of the optimal model for clinical application. Notably, compared to models reported in previous studies [35], our predictive model has included more predictors. The use of SHAP analysis improves model interpretability, which is a key advantage over “black box” machine learning models [36]. Rigorous external validation with an independent external cohort ensures the model’s generalization ability, which is critical for clinical translation [13]. Furthermore, we comprehensively evaluated calibration performance across multiple time horizons (3-, 5-, 7-, and 10-year) using calibration curves, as recommended by the TRIPOD guidelines, providing a transparent, visual assessment of probabilistic accuracy to complement discrimination metrics.

### Limitations of the study

This study has several limitations. First, the retrospective nature may lead to selection bias and impair causal inference. Second, important prognostic factors including Ki-67, detailed chemotherapy regimens, targeted therapy, and neoadjuvant therapy are missing from the SEER dataset, which may weaken model completeness. Third, this study lacks of genetic data (e.g., high-risk mutations, polygenic and polygenomic data), which could have provided additional predictive utility [37]. Fourth, the RSF model showed an overfitting signal, mainly caused by inherent characteristics of tree-based algorithms [38, 39]. Fifth, given the single Asian center for external validation, the findings may not be generalizable to other ethnic groups. Sixth, competing risk analysis was not performed, which may slightly overestimate BCSS. Seventh, deep learning survival models were not included due to potential overfitting in low-event datasets. Finally, SHAP provides partial interpretability but cannot fully eliminate the black-box nature of ensemble algorithms.

## Conclusion

Based on SEER data and an independent external validation cohort, we developed four prognostic models (RSF, LASSO-Cox, XGBoost, and GBM) to predict long-term BCSS in IBC patients. The RSF model outperformed other models in discrimination and calibration, while showing overall favorable clinical utility despite its limited net benefit for 3-year BCSS prediction in DCA. Additionally, we conducted risk stratification for the study cohort, which may help clinicians identify high-risk patients and design personalized treatment strategies. Our findings suggest that machine learning algorithms, notably RSF, boast promising prospects in future clinical research and application.

## Data Availability

Study data were derived from two sources: 1. The SEER Program repository, which is publicly available at https://seer.cancer.gov/. Researchers can access the SEER database by following the official application procedures. 2. The institutional clinical data used as the external validation cohort, which were collected from GMUCH. The human-participant study was approved by the GMUCH IRB (No. 2025041). Due to privacy policies and ethical constraints, de-identified clinical data are not publicly available. Researchers may obtain the data from the corresponding author by submitting a detailed research proposal and institutional ethical approval. Relevant descriptive statistics and key model performance metrics are available in the article and supplementary files. The corresponding author will provide the R code for model construction and SHAP analysis upon reasonable request.
